# Reproductive justice for the haunted Nordic welfare state: Race, racism, and queer bioethics in Finland

**DOI:** 10.1111/bioe.12973

**Published:** 2021-11-24

**Authors:** Tiia Sudenkaarne, Mwenza Blell

**Affiliations:** 1 Technology, Ethics and Reproduction: Controversy in the Era of Normalisation (Kone Foundation), Tampere University, Tampere, Pirkanmaa, Finland; 2 Sociology/SoSaMiRe (Academy of Finland), University of Helsinki, Helsinki, Uusimaa, Finland; 3 Rutherford Fellow and Newcastle University Academic Track Fellow (School of Geography, Politics and Sociology), Newcastle University, Newcastle upon Tyne, UK

**Keywords:** eugenics, healthcare, queer bioethics, race, racism, welfare state

## Abstract

The Nordic welfare state aims to offer universal healthcare and achieve good health, bar none. We discuss past and present moral blind spots in welfare state bioethics through reproductive justice and queer bioethics, particularly focusing on race and racism, based on ethnographic data from Finland. Globally portrayed as aspirational and mostly uninterrogated, it is crucial to have a thorough bioethical evaluation of a Nordic model informed by Black and queer perspectives. We have come to conceptualize the Finnish welfare state as haunted. We fear that the seemingly non-racial racial hygiene continues to haunt bioethics of the welfare state as structural racism. A key cause for this concern is the lack of racial awareness in public politics and the reluctance in discussing racism due to the national agenda of color-blindness. This crucially compounds to our findings that medical professionals prefer to think they operate on “purely medical” reasoning as opposed to nuanced ethical contemplation, the latter associated with “social issues” that allegedly cannot be resolved and are outside medical interest. We discuss how the bioethical aftermath of eugenics remains unresolved. Racist, classist, sexist, ableist, and cis- and het-eronormative stratification of reproduction requires a nuanced moral compass for Nordic welfare state bioethics, not “strictly medical practice.” We suggest queer bioethics as a moral theory for recalibrating this compass, joining forces with other justice movements to tackle racism in healthcare and further to interrogate racism, sexism, ableism and cis- and heteronormativity in bioethics.

## 1 Introduction

The Nordic welfare state aims to offer universal healthcare and good health, bar none, providing the societal backdrop for some of the happiest self-reporting nations. We discuss past and present moral lacunas in welfare state bioethics through reproductive justice and queer bioethics, particularly focusing on race and racism, based on ethnographic data from Finland. We consider structural racism in Finland stemming from eugenics, health nationality, and avoidance of ethical debate. It is worth noting that, despite claims to innocence,^1^ all Nordic countries (Scandinavia, that is Sweden, Iceland, Denmark and Norway, and Finland, not geographically part of Scandinavia) have unique welfare state models, due to historic, political and cultural differences. A fundament establishing a shared Nordic notion of healthcare, however, is the idea that the welfare state distributes universal healthcare and social benefits ideally, thus meeting ideally the definition of justice as equality. Despite its merits and the fact that it is globally portrayed as exceptionally aspirational, we urge ongoing thorough bioethical evaluation of the Nordic model informed by Black and queer perspectives. This paper focuses on Finland, but many of our findings resonate with the critiques of racism in health and bioethics more broadly. As Finland is perhaps the least analyzed from this viewpoint, We wish ti discuss its Nordic welfare state model as one of the Nordic welfare state models, each with their unique contexts.

We draw on material from a 6-month ethnographic study of Finland carried out by one of the authors, a Black non-Finnish woman anthropologist. During this 6-month period, the ethnographer and her family lived in eastern Helsinki and she carried out interviews and participant observation of life in Finland. In addition to observing and engaging with rhythms and patterns of everyday life as an ethnic minority mother of a young child, she focused on day-to-day interactions of people with state institutions, access to healthcare, and experiences with work and education. Interviews were carried out with Somalis living in Finland (the largest and longest-standing racialized ethnic minority group and thus the group with most shared knowledge of Finland from an ethnic minority standpoint) with different professions and a range of specific non-Somali Finnish professionals about issues relating to reproduction, gender, sexuality, and ethnicity, including doctors, academic researchers, and NGO workers. We focus on the ethnographer's fieldnotes and interviews with white Finnish informants including healthcare professionals and researchers, and Finnish Somali women to explore welfare state bioethics, particularly reproductive ethics. The study was motivated initially by the absence of people of color in writing about reproductive technologies in Finland.

This work is part of our larger research project on ethics and reproduction in Finland. We have come to conceptualize the Finnish welfare state as haunted, following Gordon, encompassing modern forms of dispossession, exploitation, and repression that concretely impact the lives of marginalized people and also our shared conditions of living, such as racial capitalism and state violence as public health policy.^2^

Based on our ethnographic findings, we suggest that the norm in Finland seems to be denial about historic and ideological connection between the welfare state and eugenics: racism, classism, sexism, ableism, and cis- and heteronormativity. The welfare state is haunted in the sense that what has been suppressed or concealed is very much alive and present, yet eerie and troubling.^3^ To consider the Finnish welfare state as haunted is to focus on its marginals, those allegedly in need of violent control and technocratic governance in the name of public health, in teasing out those singular and yet repetitive instances when the nation appears unfamiliar to those invested in the status quo and denial. In calling Finnish welfare state bioethics haunted, we seek to resolve this haunting through reproductive justice and queer bioethics, targeting racism in healthcare. We suggest that the “something to be done,” integral to Gordon's concept of haunting, in this case is a reorientation of the society's ethical approach and action to resolve the social violence that causes this haunting.^4^

We begin with sketching out what this ethical approach calls for us to no longer “block from view” including things that “are supposedly over and done with” and things whose “oppressive nature is continuously denied.”5 We then propose ways that attention to these sources of haunting and their impact can improve queer bioethics as a framework already aligned with social justice, to tackle racism in healthcare.

## 2 Health Nationality, Racial Hygiene, and Nordic Welfare State Bioethics

In Finland, conversations about race raise negative reactions and are largely avoided in public debate, with color-blindness seen as a sufficient aim.^6^ Indeed, the ban on recording of race or ethnicity in population statistics aims to keep state services color-blind and to disallow racial profiling. However, this official and popular disengagement with race ignores much theoretical and empirical research on whiteness and racial hierarchy.^7^ Many of our white Finnish informants suggested that in recent years racism has become associated with a particular right-wing political party's supporters, imagined as ignorant, compartmentalizing it to that group instead of recognizing more pervasive and structural forms of racism. Active avoidance and silencing of talk about racism as pervasive, rather than merely individual (residing in *other* individuals), can enhance structural racism.^8^ In fieldwork, white Finns often used the fact that Finns were not always considered white historically as a way to deflect claims that Finnish society had a problem with racism, as though Finns’ well-documented aspiration to become white, eventually accomplished, was not itself fundamentally racist and did not involve “the racialization of indigenous people and minorities perceived as threats to the modernizing nation.”^9^

Connections between the emergence of public health politics and racial hygiene—several practices of controlling reproduction considered to have unfavorable qualities managed by the state with the ethos improving the quality of “the national body”^10^—tend to go unacknowledged in welfare state bioethics. In Finland, people with psychological, neurological, developmental, and sensory problems or substance addictions were particularly targeted, but also poor people, working class families, ethnic minorities (the Finnish Roma and Sámi) and queer people.^11^ Its key feature is the notion that physical, psychological, social, and moral qualities are hereditary. Indeed, for much of Finnish history “[p]opulation policy, eugenics, public health and family policy have been more or less the same thing.”^12^ By the 1930s the state had invented a duty to protect itself from unfit qualities by limiting both the reproductive and political rights of their bearers (such as the right to vote);^13^ the ethos of this sterilization policy still haunts Finnish trans people today through legislation conditioning so-called gender reassignment on medical proof of sterility.^14^

The importance of hygiene as a concept for control emerged during the 18th and early 19th centuries. A political and societal issue, hygiene not only referred to cleanliness but to a ubiquitous ethos of caring for health and preventing disease. This stemmed from the tradition of Western medicine to understand health as a personal condition that requires skills to maintain.^15^ Healthcare became personal control but also population control, aiming to secure and enhance vitality. This ethos assigned medical expertise, institutions and practices with the task of maintaining social control.^16^

Once the horrors inflicted on people by the Third Reich were fully exposed after the Nuremberg trials, eugenics was reconstructed within science, reframed through health education about heredity as population health. In Finland, governmental control of unwanted “hereditary” qualities was considered rational and central to health politics for decades after the war.

The end of the Second World War had severed Finland’s historically and politically strong ties to German-speaking Middle- Europe;^17^ however, the ideological legacy of this remains fiercely protected from examination. Turning to the new Anglo-American science, Finland and other Nordic countries savored the ethos of racial hygiene by reforging it as heritage hygiene (*perintöhygenia*).^18^ Leading medical experts defined heritage hygiene as the new Nordic eugenics, “a strictly medical practice,” aiming to prevent hereditary diseases, employing a medical ethic that presented “the thinking doctor” with “an obvious duty to prevent racially unfit breeding.”^19^ For several decades in the mid-20th century, numerous medical professionals promoted a notion of society’s best interest over individual reproductive rights, including compulsory racial hygienic sterilization, which continued until 1970,^20^ with the number of such sterilizations peaking in the period 1956-1963.^21^ Medical ethics considered such sterilization rational, well founded, and justified; a neutral and sensible practice with nothing to do with violence or racism. Yet it obviously relied on the notion that a population can be evaluated based on what later became genetics.^22^ Compulsory sterilization sat alongside a host of other eugenic policies, including denying social benefits, impeding marriage, and compulsory eugenic abortions. Moreover, these policies defined minorities like the Sámi or Roma as undesirable for Finnish society, a logic that included them with other people considered inferior in a set of technocratic measures designed “to ensure the survival of ‘Us,’ the ‘people,’ in the face of the threat of the ‘Other’.”^23^ We find that the ethos of racial hygiene as a neutral, rational, subtle practice haunts Finnish healthcare, as it was never really disowned. This can be seen both in the ways Finnish doctors are empowered to reject infertile patients seeking access to assisted reproduction technologies if they are disabled, discovered in the fieldwork, and the strange absence of referrals for IVF treatment for Somali women struggling, sometimes for years, with fertility problems, evident in interviews with them. The silences around Finland’s engagement with theThird Reich’s ideology and Finland’s own longstanding eugenics policies meant that even speaking to Finnish academics about these topics during fieldwork was typically a very tense experience and featured denial about past events, unless the academic was specifically an expert on Finnish scientific racism.

Helén and Jauho look at the interplay of societal control and individual rights through the concept of health nationality (*terveys-kansalaisuus*).^24^ Health nationality includes a person in the national body, part of public health, by indoctrinating them to learn about and manage health promoting practices. Especially Nordic national projects have relied on Kantian notions of moral maturity and cultivation of moral character. This public health enlightenment establishes caring for one's health as a personal duty to be fulfilled for the nation. However, health nationality also justifies control and exclusion of those deemed unfit. These marginalized others thus become ideologically central for defining health nationality, a continuum on which individuals are defined with fuller or fewer civil rights within the population body, including subjecting the misfit to control, isolation, and various forms of care.^25^ Historically, the interplay of control, isolation, and integration operates as a governing paradigm for evaluating, defining, and interrogating the access to civil rights.^26^ Governing the vitality of people as population health subjected them to social control with health politics in the core of the processes that were to produce the structures of the welfare state.^27^

Finnish public healthcare institutionalized between 1920 and 1960, crucially connected to the construction work of Finland as a new nation. Public health was deemed to be in the best interest of the state and for the welfare of the nation, maintained with control and authority. In human rights movements emerging since the 1960s, health as a positive, social right began to challenge this, deeming every member of the national body to have the right to healthcare. Social rights and welfare emerged into Finnish public debate later than in, for example, fellow Nordic country Sweden.^28^ Health nationality includes the idea of health as a right, albeit right continues to be an elusive concept.

Public health politics and health nationality pre-exist the welfare state and both are embedded in the construction of the welfare state. A key function of a Nordic welfare state is to manage risks related to the human condition throughout one's life span, from birth to eldercare, with a democratic, centralized system of redistribution and governance. Individual rights and duties are part of collective processes often with legislative justifications. Another key function is to reduce inequality. Nordic welfare state health politics controls, partly provides and governs social and healthcare as a constitutional right but also monitors perceived hazards (such as the consumption of alcohol). Moreover, Nordic welfare state health politics obliges individuals to enhance their own health.^29^ Bioethics of the modern welfare state combines health nationality and public health politics with democratic, legal governance and funding of health as a social right while also subjecting individuals to various forms of control.

Since the 1990s, welfare state ideology has been accompanied by neoliberal new public management. After two recessions, cuts and reforms have not deconstructed the institutional basis of the Finnish welfare state:^30^ following a Nordic model, it has a market driven economy, but the state redistributes tax revenue to provide public health and social services. The model leans partly on social democratic principles (as is more prominently the case in Sweden and Denmark), but also on moderate conservatism and Lutheran ideals.^31^ From the viewpoint of health nationality, the welfare state under new public management emphasizes caring for health as one's personal duty through an ethos of prioritization, including notions of “self-induced illness,” such as associating obesity with ignoring that duty.^32^ As the universal healthcare promised by the welfare state is in fact stratified for minorities and marginalized groups, “universal” healthcare does not manage to produce universally good results throughout the population nor does it serve all people in the territory, connecting to various other forms of inequalities.^33^ This stands in stark contrast to social rights as guaranteed access to social goods distributed by the state as welfare through public social and healthcare services universally. Moreover, people report having to fight for those rights.^34^ We found that unemployed people face particular challenges in healthcare access and there are well-documented geographic in-equalities particularly affecting reproductive care.^35^ Moreover, people can be formally excluded from those rights, either based on hauntings of racial hygiene (e.g., trans people) or based on denying residence or citizenship to people.

## 3 Reproductive Justice, Racism, and Nordic Welfare State Bioethics

The idea that eugenics in Finland is not racist, classist, and immoral but a neutral medical practice is absurd, but we consider this to be explained partly by the lack of understanding in public policy of class or race as enduring structures. We suggest that denial that there is discrimination in Finnish society further plays into prioritization logics of the welfare state under new public management, building a new health nationality hierarchy based on employment status, race, disability, and gender and sexual variance.

As we have outlined, part of the control politics of Nordic welfare state bioethics has always been about reproductive control with structural normativities justified by racial hierarchy either organized by presumed genetic traits, nationality (access to citizenship), or direct racism. As we have mentioned, in Finland concerns about reproduction led to both positive and negative programs of eugenics, positive that encouraged those with desirable traits to produce superior people and negative to eliminate inferior people from future generations. Within these logics class and race were connected in that Finnish working-class people were considered to belong to the “biologically lowest level” so the aims were to promote reproduction among white middle class Finns and to limit the possibility of the same for all others.^36^ This is because eugenicists framed their arguments not only in terms of improving the race, but also in terms reducing the cost of subsidizing the unfit. One of the greatest worries of the 1930s eugenicists was that the least fit appeared to have increased fertility while the socially desirable classes experienced a decline in their birthrate.^37^ During fieldwork, one white Finnish woman told the ethnographer she had been congratulated by a stranger for being pregnant and white, with the stranger suggesting there was a zero sum game at play, where “others” clearly reproduced too much comparatively. This idea that racialized others reproduce too much was echoed in the account of a Somali woman who reported that midwives laughed as she left the hospital with her newborn infant saying “see you next year” implying she would give birth again in a year's time, which was not her intention.

These hauntings also occur in the public sphere. In 2017, the then-Prime Minister Antti Rinne publicly bemoaned the low birth rate of Finns and called for women to engage in a “birthing bee” (*synny-tystalkoot*), combining the tradition of communal labor or neighbor help with the nationalist slogan of the post-war era of reproducing “for the country.” Whilst some noted at the time that this echoed Nazi slogans, it also echoed the propaganda of Finland's own Väes-töliitto (the Finnish Population and Family Welfare League), an influential NGO, formed in 1941, in its days of explicit eugenic ideology when it propounded the notion that reproduction was a civic responsibility rather than a private matter (although it only meant this to apply to abled, middle class white Finns).^38^ Privileged racial identity gives whites a powerful incentive to preserve the existing social order intact.^39^ Indeed, the idea the (white, middle class) status quo in Finland is in need of protection is very resonant from the fieldwork, seemingly justifying an unwillingness to look at the problems we have outlined.

To Roberts, attention to race can help redefine reproductive liberty in a way that accounts for its importance to human dignity and equality. On this note, the ethos and ethical underpinnings of the strong welfare state must be exorcised of their eugenic hauntings.

Racial disparity in access to reproductive technologies is gaping. The impact race has on “the right to create children” with reproductive technologies makes people of color likely to serve as a bioresource for reproductive markets catering to the white middle class.^40^ Reproductive technologies tend to be more conforming than liberating and often reinforce the status quo rather than challenge it. Most often they complete a traditional nuclear family by providing a white abled affluent cis- and heteronormative married couple with a matching child.^41^ Our health professional informants either had witnessed or expressed concerns about providing services to single women, queer people, and people with disabilities even though legislation in Finland clearly grants access and offers protection against discrimination on the grounds of race, gender^42^ and gender and sexual variance.

Globally, poor women of color are the most vulnerable to reproductive control. In the USA, Black people make up a disproportionate number of infertile people not using reproductive technologies^43^ and the profile of people most likely to use IVF is precisely the opposite of those most likely to be infertile. The use of high-tech fertility treatment does not depend on the physical ability to have a child. Instead, the racial disparity is a result of not only cultural preferences but financial barriers and more deliberate professional manipulation.^44^ It seems people of color face barriers to accessing them in Finland, too. Our preliminary findings with Somali women indicate that experiences of reproductive healthcare vary greatly between women from complete satisfaction to feeling like “the doctor didn't want to waste time on me.” Somali women expressed the feeling that Finnish people disapproved of their preference for families with more than one or two children, similar to previous findings.^45^ Medical professionals also seemed unaware of the stratified effect of race in accessing reproductive technologies. Few could recollect ever having treated people of color for infertility and they had not noticed this before being asked to reflect on it.

As already discussed the preoccupation with (white) Finns rate of reproduction continues, in popular media, in politics, and in daily life. These discourses continue the prior logics from the era of explicit eugenics by treating as very important the question of whether Finns are “dying out” because of not having enough children, whilst ignoring the fact that birth rates of non-white Finns are relatively high. The perspective that it is white Finns whose reproduction is important to worry about is part of how reproduction of people other than white Finns is constructed as a threat. The sense that ethnic minorities are not Finnish and rather than contributing they take away from the welfare state that only Finns have a right to was explained thusly by one informant: “[the welfare state] is a ready-made thing and who are you and what have you contributed?… the state invests in every child so much money that it is way past the child is 30 or 40 until the debt has been paid.” As Bergenheim has pointed out, from this perspective “[t]he productive people, the society and the nation were one and the same.”^46^ This exclusionary way of thinking about who has rights to the goods offered by the welfare state, along with the long history of pathologizing reproduction among any but abled, middle-class cis heterosexual white Finns may form part of the unspoken justification for failing to refer non-white Finns with fertility disruptions for high-tech treatments like IVF.

From a bioethical viewpoint, racial steering is frequently dressed in medical garb,^47^ as is cis- and heteronormative steering against queer people and single women. When discussing reproductive technologies for female couples, single women, or trans people with our informants, we found doctors associated medical indication with ethical streamlining and contrasted it with “social” reasons that entailed moral ambiguity. Yet the very diagnosis of infertility depends on social factors. Some researchers have linked the contrasting response of infertile Black American women to their spiritual or psychological outlook on adversity; they may be more likely to attribute it to faith or god's will than seek to address it in science.^48^ This was also suggested by some of our Somali women informants, although many of them also described seeking medical help for gynecological and reproductive issues (with varying satisfaction). Some reported only having received proper medical attention outside Finland.

Why are people of color underrepresented among users of reproductive technologies in a Nordic welfare state, supposedly distributing welfare through public social and healthcare services invariably? We suggest this is due to structural racism, stemming from an unresolved past ethos of racial hygiene as a “strictly medical practice” that understands itself to be rational and neutral, combined with past and present health nationality politics and a tendency to prefer the status quo over ethical debate. A parallel that may be useful in understanding how structural barriers function in Finnish reproductive medicine is in the recent complete barrier for female couples in accessing IVF in the public healthcare system, even in cases with clear medical indication of infertility. The Act on Assisted Reproductive Treatment granted female couples and individual women the right to treatment in the public system in 2007. However, the Non-Discrimination Ombudsman, one of the highest officials on equality in Finland, noted in 2016 that medical directors of public university hospitals had argued that there was a need to “prioritize due to insufficient resources” and set a standard policy of refusing treatment to female couples and individual women.^49^ In our interviews, conducted 3 years after the Ombudsman had publicly explicitly deemed this practice discriminatory, IVF doctors admitted that those in charge of the public system chose to quietly close down existing donor sperm services, thus denying heterosexual couples access to sperm as well, in order to avoid offering donor sperm to same-sex female couples, under the guise of saving resources (both welfare state healthcare costs and donor gametes). They contemplated very little the ethical infringement resulting from the nexus of permissive legislation and discriminatory practice, even though the Ombudsman had claimed it “unbelievable that the public health care has lived up to the discriminatory guideline by the medical directors against the will of the lawmakers.”^50^ Donor gamete biobanks have since been established within the public sector, with the first babies born in 2020. The fear of many medical professionals that access for female couples and individual women would negatively affect the availability of donor gametes has been proven wrong: many donors have expressed their desire to help such patients particularly.^51^

We fear that the seemingly non-racial racial hygiene, later thinly veiled as health concerns under new eugenics, continues to haunt the bioethics of the welfare state as structural racism. A key cause for this concern is the lack of racial awareness in public politics and the inability to talk about racism. This crucially compounds to our findings further reflect that medical professionals prefer to think they operate on “purely medical” reasoning as opposed to nuanced ethical contemplation, the latter associated with “social issues” that allegedly cannot be resolved and are outside medical interest. Access to reproductive technologies without medical indication remained a controversial issue to many of our informants, despite there being distinct legislative will to endorse it for years. Further, concentrating the power of population enhancement in the hands of the socially privileged exacerbates differences in the status and welfare of social groups.^52^

In Nordic welfare state bioethics, health is politicized not only through official authority, legislation and institutionalized practices but crucially, politics of health is practiced by social welfare and healthcare practitioners, particularly by medical doctors.^53^ This includes normative judgment in everyday care practices, systems, and policies.

In the interviews, we repeatedly encountered a jarring split between “medical” and “social” in framing ethics, as if all true moral contemplation would take place in the latter and thus be irrelevant to the former. Informants willing to partake in ethical debate had experienced dismissal or downright aggression in situations where medical ethics was under scrutiny by non-doctors. It was also suggested that public debate on bioethical fundaments like euthanasia is discouraged by medical organizations as public opinion might swing politics against “the medical opinion.” Several doctors were notably better attuned to technical developments within medicine than to biomedical ethical issues, those of race and racism in healthcare invisible to them. Many informants noted that ethical training for medical doctors in Finland is a one-course event at best, their ethical professionalism relying on the notion that good doctors make good decisions. Associating ethical debate, which usually requires admitting that there might be something wrong with a practice, with not being a good doctor is remarkably unsophisticated and obviously bioethically grave. The notion that claiming something as medical excludes it from the realm of moral contemplation is engraved in Finnish medical ethics, introduced in its professional form by eugenicist Palmén who set every “thinking doctor” with the “obvious duty to prevent racially unfit breeding.” Palmén's thinking doctor has evolved into today's good doctor who sees no moral flaw in protecting business as usual.^54^ Haunted by the unresolved ethos of eugenics, the good doctor performs prioritization to protect welfare state resources under new public management.

## 4 Reimagining A Nordic Welfare State Bioethics: Queer Bioethics as A Moral Theory

What haunts Nordic welfare state bioethics is the historic non-interruption of racial hygiene: it was deemed an issue of “strictly medical practice” and thus required a strictly medical solution, which was to eventually stop the blatant forms of such practice. However, the bioethical aftermath remains unresolved. Racist, classist, sexist, ableist, and cis- and heteronormative stratification of reproduction requires a nuanced moral compass for Nordic welfare state bioethics, not “strictly medical practice.” We offer queer bioethics to recalibrate this compass, with apologies that the gargantuan nature of the effort falls outside the scope of this paper.

Queer bioethics, formulated by Lance Wahlert and Autumn Fiester, builds on medical humanities and on a critical approach toward politics of medicine. Queer bioethics promotes active involvement in debate on the ethics and moral conceptions in medicine and biosciences.^55^

Queer bioethics aims to highlight the political aspects present in formulation of any ethical principles by unlocking historic contexts and complex dependencies that usually go undetected in bioethical inquiry. Injecting bioethical debates with awareness of normative power and their effects on people whose experiences and existence do not comply with normativities, a cornucopia of enhanced human flourishing becomes imaginable.^56^ Queer thinking that critically addresses the complexities of normativity is needed for making changes, understanding diversity, dismantling injustice and enhancing justice in welfare state bioethics. To Wahlert and Fiester, queer bioethics challenges the politics of normativity and reveals discriminative and unjust practices in healthcare and the presumptive legitimacy of the normative.^57^ Finally yet importantly, they define queer bioethics to serve as a moral theory.^58^

To clarify, haunting and queer bioethics as a moral theory are two independent concepts, but can most certainly be deployed together. Hauntings are results of violence: eradication, marginalization, and oppression either through action or omission. Resolving hauntings through queer bioethics as a moral theory could contribute to developing more complex analysis of intersectionality as a form of critical inquiry and praxis that resists violence.^59^ Crucially, this requires placing gender and sexual variance at the core of ethical discussions,^60^ as cis- and heteronormativity continue to be downplayed despite their “consistently perverse, violent, and demeaning” effects, “turning people into animals and turning white women into reproducers of the (white) race and the (middle or upper) class.”^61^ There currently is no moral theory with this normative component.

We suggest the collective project for building queer bioethics as a moral theory to galvanize justice-oriented inquiries in bioethics.^62^ To us, such a project can reimagine the ethos of the welfare state, explicitly interrupting eugenic legacy of racism, sexism, classism, ableism and cisand heteronormativity. Queer bioethics as a moral theory could offer an avenue for justice movements to work together in tackling race and racism in healthcare, as justice is a central concept in queer bioethics; moreover, it is a bioethical principle often ignored in principlist approaches.^63^ Queer bioethics as a moral theory should contribute to intersectionality true to its origins in Black feminism^64^ by using an analysis of violence as a navigational tool for developing an intersectional understanding of power and justice.^65^ As Black feminist scholar Hill Collins has pointed out,

ostensibly colourblind rules and regulations reinscribe social inequality as firmly as the use of force. In this context, violence did not disappear. Instead, it became embedded in the rules, and became even more routinized via a system of seemingly non-discriminatory ideas and practices. State-sanctioned violence that is not defined as violence at all, yet that is essential in sustaining racial inequality persists, seemingly hidden in plain sight…. Collectively, these seemingly disparate expressions of violence constitute a malleable conceptual glue that both structures the forms that violence takes within distinctive systems of power and that facilitates their smooth interaction. In this sense, violence constitutes a saturated site of intersectionality where intersecting power relations are especially visible.^66^

Queer bioethics as a moral theory can thus help to interrogate white supremacy, conflation of whiteness with normality, and disrespect for ethnic diversity by unearthing ethnic bias and marginalization. It can provide a framework to investigate whether a case allows infringements of the bioethical principle of justice based on race, gender, gender and sexual variance, or ability. As a moral theory, it needs to provide ethically sustainable answers to cultural relativism in healthcare harmful to queer people of color. It needs to offer powerful counterstories to cultural imperialist narratives, for example, assumptions that a white queer person is more autonomous than a queer person of color who makes sense of their queerness outside the LGBTQI+ human rights agenda. Ultimately, queer bioethics as a moral theory can contribute to disempowering racialized stereotypes related to health, such as the assumption that Black women are more fertile than white women.

Queer bioethics as a moral theory must also offer contributions particularly to reproductive justice, another important Black feminist concept. A crucial queer and transgender viewpoint to stratified access to reproductive justice has been to link current trans treatment practices to eugenic sterilization practices and to discuss, how within Nordic welfare state pronatalist agendas, the declining birth rate entails white, middle-class heterosexual responsibility to reproduce for the sake of the nation.^67^ However, queer reproduction should not become part of a homonationalist agenda. Queer bioethics as a moral theory should incorporate reproductive justice as the human right to have access to contraception and safe abortion, as the right to have children, as the right to not have children, and the right to parent the children one has in safe and sustainable communities. A queer feminist framework's approach to principles, informed by queer bioethics, feminist bioethics, and Black feminist thought, could also poignantly challenge the definition and application of the principle of justice, for example considering reproductive technologies to offer ethical possibilities sustainable for both the affluent white gay couple and the woman of color serving as their transnational surrogate. As study of racism in healthcare and bioethics suggests, queer people of color are rendered to several vulnerabilities, and their particularities in the Finnish welfare state context require dedicated research beyond the scope of this paper.

## 5 Conclusion

Justice and injustice are crucial in looking at the historic development of Finnish healthcare and health politics. The welfare state bioethics as equal distribution of health as a social right must be interrogated for past and present instances of marginalization and for current structural racism as showing the work of intersecting oppressions that continue to haunt the Finnish welfare state. Such hauntings show up in everyday violence that is embedded in the status quo of the health system and medical practice. Welfare state bioethics must also revisit the notion of health nationality and resolve to remain attentive to forms of social violence in the past and present that others might rather not talk about, whether they be sterilizations or unequal access to healthcare. It is essential that this and not the medical versus social, status quo ethics perspective be part of medical training and that Finnish society more broadly develop this kind of ethical sensitivity in order to become a more just society. We further encourage developing queer bioethics as a moral theory to join forces with other justice movements to tackle racism in healthcare and further to interrogate racism, classism, sexism, ableism and cis- and heteronormativity in bioethics.

## Footnotes

1 Keskinen, S., Tuori, S, Irni, S., & Mulinari, D. (Eds.). (2009). *Complying with colonialism: Gender, race, and ethnicity in the Nordic region.* Ashgate.

2 Gordon, A. F. (2011). Some thoughts on haunting and futurity. *Borderlands,* 10(2), 1–21, p. 2.

3 Ibid.

4 Ibid.

6 Keskinen, S., Seikkula, M. K., & Mkwesha, F. (Eds.). (2021). *Rasismi, valta ja vastarinta:* *Rodullistaminen, valkoisuus ja koloniaalisuus Suomessa.* Gaudeamus.

7 Puuronen, V. (2011). *Rasistinen Suomi*. Gaudeamus, p. 29.

8 Vuolajärvi, N. (2014). Rotu etnisten suhteiden tutkimuksessa. In S. Irni, M. Meskus, & V. Oikkonen (Eds.), *Muokattu elämä: teknotieteen ja materiaalisuuden muunnelmat* (264–303, p. 265). Vastapaino.

9 Keskinen, S. (2019). Intra-Nordic differences, colonial/racial histories, and national narratives: Rewriting Finnish history. *Scandinavian Studies, 91(1–2),* 163–181, p. 164.

10 Kananen, J. K., Bergenheim, S. M. C., & Wessel, M. (Eds.). (2018). *Conceptualising public health: Historical and contemporary struggles over key concepts.* Routledge.

11 Mattila, M. (2005). Sterilointipolitiikka ja romanit Suomessa vuosina 1950-1970. In A. Häkkinen, P. Pulma, & M. Tervonen (Eds.), *Vieraat kulkijat—tutut talot: Näkökulmia etnisyyden ja köyhyyden historiaan suomessa* (pp. 402–452). SKS; Ranta, K., & Kanninen J. (2019). *Vastatuuleen: Saamen kansan pakkosuomalaistamisesta*. S&S Kustannus; Honkasalo, J. (2019). In the shadow of eugenics: Transgender sterilization and the struggle for self-determination. In R. Pearce, I. Moon, K. Gupta, & D. Lynn Steinberg (Eds.), *The emergence of trans: Cultures, politics and everyday lives* (pp. 17–33). Routledge; Bergenheim, S. (2018). Cherishing the health of the people: Finnish non-governmental expert organisations as constructors of public health and the ‘people’. In J. Kananen, S. Bergenheim, & M. Wessel (Eds.), *Conceptualising public health: Historical and contemporary struggles over key concepts* (pp. 101-118). Routledge.

12 Bergenheim, S. M. C. (2017). *The population question is, in short, a question of our people’s survival: Reframing population policy in 1940s Finland.* Proceedings of the 29th Congress of Nordic Historians, Aalborg, August 15–18, p. 112.

13 Mattila, M. (2003). Rotuhygienia ja kansalaisuus. In I. Helén, & M. Jauho (Eds.), *Kansalaisuus ja kansanterveys* (pp. 109–142, p. 110). Gaudeamus.

14 Honkasalo, op. cit. note 11.

15 Helén I., & Jauho, M. (2003). Johdanto. In I. Helén & M. Jauho (Eds.), *Kansalaisuus ja kansanterveys* (pp. 7–8). Gaudeamus.

16 Ibid: 7.

17 Meskus, M. (2009). *Elämän tiede*. Vastapaino, p. 55.

18 Ibid: 56–57.

19 Palmén, A. J. (1956). *Lääkärin etiikka*. WSOY; see also Meskus, op. cit. note 17, p. 57. English translation by authors.

20 Ibid.

21 Mattila, op. cit. note 13; Bergenheim, op. cit. note 11.

22 Meskus, op. cit. note 17; cf. also Palmén, op. cit. note 19.

23 Bergenheim, op. cit. note 11, p. 116.

24 Helén, I., & Jauho, M. (2003). Terveyskansalaisuus ja elämän politiikka. In I. Helén & M. Jauho (Eds.), *Kansalaisuus ja kansanterveys* (pp. 13–32, p. 15). Gaudeamus.

25 Ibid.

26 Mattila, op. cit. note 11, pp. 402–452, p. 110.

27 Ibid: pp. 402–452.

28 Ibid: pp. 402-452.

29 Saari, J. (2020). *Samassa veneessä: hyvinvointivaltio eriarvoistuneessa yhteiskunnassa*. Doc-endo, pp. 46–47.

30 Ibid.

31 Takala, T., & Häyry, M. (2019). Research ethics and justice:The case of Finland. *Cambridge Quarterly of Healthcare Ethics,* 28(3), 551–576, p. 16.

32 Helén & Jauho, op. cit. note 24, p. 31.

33 Rahkonen, O., & Lahelma, E. (2009). Terveys hyvinvointivaltiossa: Peter Townsendin tulkinta terveyden eriarvoisuudesta. In J. Saari (Ed.), *Hyvinvointivaltio: Suomen mallia analysoimassa* (pp. 263–283, p. 273). Gaudeamus.

34 Ibid.

35 Huotari, T., Rusanen, J., Keistinen, T., Lähderanta, T., Ruha, L., Sillanpää, M. J., & Antikainen, H. (2020). Effect of centralization on geographic accessibility of maternity hospitals in Finland. *BMC Health Services Research, 20,* 1–9.

36 Hämäläinen, P. (1985). Suomenruotsalaisten rotukäsityksiä vallankumouksen ja kansa- laissodan aikoina. In A. Kemiläinen (Ed.), *Mongoleja vai germaaneja? Rotuteorioiden suomalaiset* (pp. 407–420). Suomen Historiallinen Seura.

37 Roberts, D. (2017). *Killing the black body*. Vintage Books, pp. 215–216.

38 Nyman, R. (2017, August 23). Antti Rinne kutsui suomalaisia synnytystalkoisiin. *Iltalehti*, Retrieved February27, 2021,from https://www.iltalehti.fi/politiikka/a/201708232200346185; Bergenheim, op. cit. note 11.

39 Roberts, op. cit. note 37, pp. 243–244.

40 Ibid: 246; Bailey, A. (2011). Reconceiving surrogacy: Toward a reproductive justice account of surrogacy. *Hypatia,* 26(4), 715-741; Homanen, R. M. P. (2018). Reproducing whiteness and enacting kin in the Nordic context of transnational egg donation: Matching donors with cross-border traveller recipients in Finland. *Social Science & Medicine, 203,* 28–34.

41 Ibid: 247-248; Mamo, L. (2007). *Queering reproduction: Achieving pregnancy in the age of technoscience*. Duke University Press; Kantsa, V., Zanini, G., & Papadopoulou, L. (Eds.). (2015). *Fertile citizens: Anthropological and legal challenges of assisted reproduction technologies*. Alexandria Publications.

42 By differentiating between gender and gender and sexual variance we aim to facilitate research on cis-gendered normativities, material realities and injustices.

43 Roberts, op. cit. note 37, pp. 250–252.

44 Ibid: 253.

45 Degni, F., Pöntinen, S., & Mölsä, M. (2006). Somali parents’ experiences of bringing up children in Finland: Exploring social-cultural change within migrant households. *Qualitative Social Research,* 7(3), 1–16.

46 Bergenheim, op. cit. note 12, p. 133.

47 Roberts, op. cit. note 37, p. 255.

48 Roberts, op. cit. note 37, pp. 259–260.

49 Non-Discrimination Ombudsman. (2016). *Access to fertility treatment has to be granted on an equal basis also in public health care: Denying service provision due to sexual orientation violated the Non-Discrimination Act.* Retrieved February 25, 2021, from https://syrjinta.fi/en/-/access-to- fertility-treatment-has-to-be-granted-on-an-equal-basis-also-in-public-health-care-denying-service-provision-due-to-sexual-orientation-violated-the-non-discrimination-act. The case has recently been considered by the Supreme Administrative court.

50 Ibid.

51 Lukkari, M. (2020). *Julkisen hedelmöityshoidon ensimmäiset lahjasolulapset ovat syntyneet*. Retrieved February 25, 2021, from https://yle.fi/uutiset/3–11677762

52 Roberts, op. cit. note 37, p. 283.

53 Helén & Jauho, op. cit. note 24, pp. 19–20.

54 Meskus op. cit. note 19, p. 57.

55 Wahlert, L., & Fiester, A. (2012). Queer bioethics: Why its time has come. *Bioethics,* 6(1), i-iv; Wahlert, L., & Fiester, A. (2014). Repaving the road of good intentions: LGBT health care and the queer bioethical lens. *Hastings Center Report, 44(4),* S56–S65.

56 Nelson, J. L. (1998). The silence of the bioethicists: Ethical and political aspects of managing gender dysphoria. *GLQ, 4(2),* 213-230; Nelson, J. L. (2012). Still quiet after all these years: Revisiting “The silence of the bioethicists.” *Journal of Bioethical Inquiry, 9,* 249–259.

57 Wahlert & Fiester (2012), op. cit. note 54, pp. ii–iv.

58 Ibid.

59 Hill Collins, P. (2017). On violence, intersectionality and transversal politics*. Ethnic and Racial Studies, 40*(9), 1460–1473.

60 cf. Wahlert & Fiester (2012), op. cit. note 54, p. iii.

61 Lugones, M. (2007). Heterosexualism and the colonial/modern gender system. *Hypatia,* 22(1), 186-209, p. 202.

62 Obviously, the task exceeds the scope of this article.

63 On justice unused as a principle, see e.g., Donchin, A. (2001). Understanding autonomy relationally: Toward a reconfiguration of bioethical principles. *Journal of Medicine and Philosophy, 26*(4), 365–386.

64 cf. Nash, J. C. (2018). *Black feminism reimagined: After intersectionality*. Duke University Press.

65 Hill Collins, op. cit. note 58, p. 1464.

66 Ibid: 1464.

67 Honkasalo, J. (2018). Unfit for parenthood? Compulsory sterilization and transgender reproductive justice in Finland. Journal of International Women's Studies, 20(1), 40–52.

